# Human UTY(KDM6C) Is a Male-specific *N*^ϵ^-Methyl Lysyl Demethylase*

**DOI:** 10.1074/jbc.M114.555052

**Published:** 2014-05-05

**Authors:** Louise J. Walport, Richard J. Hopkinson, Melanie Vollmar, Sarah K. Madden, Carina Gileadi, Udo Oppermann, Christopher J. Schofield, Catrine Johansson

**Affiliations:** From the ‡ Chemistry Research Laboratory, Department of Chemistry, University of Oxford, Mansfield Road, Oxford OX1 3TA, United Kingdom,; the § Structural Genomics Consortium, University of Oxford, Headington OX3 7DQ, United Kingdom, and; the ¶ Botnar Research Centre, Oxford Biomedical Research Unit, Oxford OX3 7LD, United Kingdom

**Keywords:** Chromatin, Dioxygenase, Epigenetics, Histone, Histone Methylation

## Abstract

The Jumonji C lysine demethylases (KDMs) are 2-oxoglutarate- and Fe(II)-dependent oxygenases. KDM6A (UTX) and KDM6B (JMJD3) are KDM6 subfamily members that catalyze demethylation of *N*^ϵ^-methylated histone 3 lysine 27 (H3K27), a mark important for transcriptional repression. Despite reports stating that UTY(KDM6C) is inactive as a KDM, we demonstrate by biochemical studies, employing MS and NMR, that UTY(KDM6C) is an active KDM. Crystallographic analyses reveal that the UTY(KDM6C) active site is highly conserved with those of KDM6B and KDM6A. UTY(KDM6C) catalyzes demethylation of H3K27 peptides *in vitro*, analogously to KDM6B and KDM6A, but with reduced activity, due to point substitutions involved in substrate binding. The results expand the set of human KDMs and will be of use in developing selective KDM inhibitors.

## Teaser

**Background:** UTY(KDM6C) has been reported previously to be inactive as a histone demethylase.

**Results:** Crystallography reveals that the fold of the UTY(KDM6C) catalytic domain is highly conserved with those of KDM6A/B. UTY(KDM6C) catalyzes demethylation of *N*^ϵ^-methylated lysine histone peptides at Lys^27^.

**Conclusion:** UTY(KDM6C) is a lysine demethylase that shows high structural similarity with KDM6A/B.

**Significance:** UTY(KDM6C) is a functional *N*^ϵ^-methyl lysine demethylase.

## Introduction

Lysine histone *N*^ϵ^-methylation is a ubiquitous post-translational modification that can signal either for transcriptional repression or activation in a site- and context-specific manner. Methyl group addition is catalyzed by the lysine methyltransferases, and its removal is catalyzed by members of either or both of two histone lysine demethylase (KDM)^3^ families (1). The largest family of KDMs comprises the Jumonji C (JmjC) enzymes, which are 2-oxoglutarate (2OG)-dependent oxygenases. The smaller family of lysine-specific demethylases are also oxidizing enzymes, but they belong to the flavin adenine dinucleotide (FAD)-dependent amine oxidase superfamily. JmjC KDMs can catalyze removal of all three methylation states of lysine *N*^ϵ^-methylation from most (but not all) known methylated histone lysine residues, with methylation state and residue selectivity varying between members (2–4). The JmjC KDMs require ferrous iron as a cofactor and use 2OG and oxygen as co-substrates and produce formaldehyde, succinate, and carbon dioxide as co-products (5).

Pioneering work on the KDM6 JmjC KDM subfamily identified two of the three members, KDM6A (UTX) and KDM6B (JMJD3), as histone 3 lysine 27 tri- and dimethyl (H3K27Me) demethylases (6–10). Despite sharing >88% similarity with its X chromosome-linked homologue, KDM6A/UTX (ubiquitously transcribed tetratricopeptide repeat protein on the X chromosome), the third Y chromosome-linked family member, UTY(KDM6C) (ubiquitously transcribed tetratricopeptide repeat protein on the Y chromosome), is reported not to have KDM activity (7, 9). Like KDM6A, the UTY(KDM6C) gene manifests in multiple splice isoforms (11). UTY(KDM6C) is a minor histocompatibility antigen that may induce graft rejection of male stem cell grafts (12). Both UTY(KDM6C) and KDM6A form part of the MLL3-MLL4 H3K4 methyltransferase complex, which also contains RbBP5, ASH2L, and WDR5 (8, 13).

H3K27 methylation is tightly linked to gene regulation (14). The gene promoters of repressed chromatin are enriched in K27Me_2_ and K27Me_3_, whereas in the gene bodies of active regions of chromatin, K27Me_1_ is found (15). By removing methylation at H3K27, KDM6 family members regulate transcription; for example, human KDM6A is involved in *HOX* gene regulation during development, with importance in body patterning on the anterior-posterior axis (6), and in mice, KDM6A activates expression of female-specific *RHOX* genes involved in sexual reproduction (16).

**FIGURE 1.**
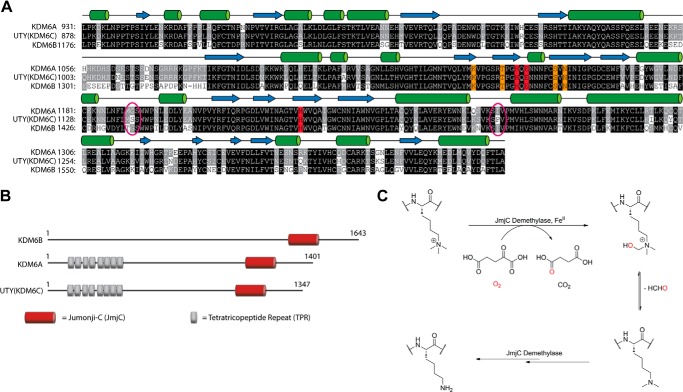
**Comparison of the members of the KDM6 subfamily of histone demethylases.** *A*, alignment of the JmjC “catalytic” domains of KDM6A, UTY(KDM6C), and KDM6B reveals a high degree of sequence identity. Residues in *black* are conserved between all three members of the human KDM6 subfamily, whereas those in *gray* are shared only by two of the subfamily. *Red*, conserved Fe(II) binding residues; *orange*, 2OG-binding residues. The crystallographically observed secondary structure for KDM6A (PDB code 3AVR) (30) is shown *above* the alignment, with α-helices as *green cylinders* and β-strands as *blue arrows*. Selected residues that differ significantly between UTY(KDM6C) and KDM6A/B are *circled* in *pink. B*, domain organization of the KDM6 subfamily. UTY(KDM6C) isoform 3 is shown. *C*, schematic of the conserved mechanism of histone demethylation as catalyzed by the JmjC KDM.

Sequence alignments predict that KDM6A/UTY(KDM6C) have similar domain organizations (Fig. 1, *A* and *B*), with N-terminal tetratricopeptide repeat domains of unknown function and C-terminal JmjC and zinc binding domains (although not assigned in previous studies, sequence analysis suggests that KDM6B may also contain tetratricopeptide repeat-like domains). Within the catalytic JmjC domains, KDM6A and UTY(KDM6C) share >96% similarity; KDM6B is less similar, sharing only ∼80%. UTY(KDM6C) contains all three of the predicted iron-binding residues as well as those predicted to be important in 2OG binding (Fig. 1*B*).

There is evidence that the biological roles of KDM6A and UTY(KDM6C) extend beyond H3K27Me_3_-related demethylation. KDM6B and KDM6A regulate transcription in a non-catalytic manner via their interactions with T-box proteins (17, 18). In addition, KDM6A and UTY(KDM6C) are capable of H3K27 demethylase-independent gene regulation (*e.g.* in regulation of Fnbp1 expression, which is thought to be mediated by alteration of H3K4 methylation levels), and both are suggested to have distinct functions in mesoderm development (13, 19, 20). Recent work reveals a sex-dependent effect after KDM6A knock-out in embryonic stem cells and mice. Some male KDM6A-deleted littermates survive to birth, whereas in the females, KDM6A deletion is embryo-lethal (13, 21, 22). Interestingly, KDM6A is also known to escape X chromosome inactivation, resulting in a dosage imbalance between males and females (23). Down-regulation of UTY(KDM6C) is associated with an increased risk of male cardiovascular disease (24).

There is thus interest in defining the roles of KDM6A and UTY(KDM6C) from both the basic science and therapeutic perspectives. Despite the reports that UTY(KDM6C) is not an active KDM, the combined sequence analyses and cellular results suggested to us that, like KDM6A, UTY(KDM6C) might indeed be a functional KDM. To investigate this possibility, we produced the catalytic and zinc-binding domains of UTY(KDM6C) and characterized them by crystallography and turnover assays. The results reveal that UTY(KDM6C) possesses KDM activity but, at least when tested *in vitro* in recombinant form, at a substantially lower level than for KDM6A/B. This is, at least in part, due to substitution of an isoleucyl residue in KDM6B and KDM6A for a prolyl residue in UTY(KDM6C). UTY(KDM6C) activity is also inhibited by small molecule probes developed as KDM6B inhibitors (25). The finding that UTY(KDM6C) is a functionally active KDM therefore has consequences both for its biological role and in interpreting the results of small molecule studies targeting KDMs.

## EXPERIMENTAL PROCEDURES

### 

#### 

##### Protein Constructs

DNA encoding for the JmjC domain and zinc binding domain of human UTY(KDM6C) (residues Pro^818^–Ser^1347^, isoform 3, GI: 33188431) was amplified from a clone kindly provided by Kai Ge (Addgene plasmid 17439) (7) and inserted into a pNH-TrxT vector (GenBank^TM^ accession number GU269914). DNA encoding for the full-length protein (isoform 3) was amplified from the same plasmid and inserted into a pcDNA3-N-FLAG-LIC vector for mammalian expression. Constructs encoding regions Leu^878^–Ser^1347^ and Ser^840^–Ser^1347^ of UTY(KDM6C) were amplified from an Origene cDNA clone (isoform 3) and cloned into a pFastBac-derived vector containing a tobacco etch virus protease-cleavable C-terminal His_10_ tag. KDM6B and KDM6A plasmids were used as described previously (25–27). UTY(KDM6C) variants were generated using the QuikChange^TM^ site-directed mutagenesis kit (Stratagene), and mutations were confirmed by DNA sequencing.

##### Protein Expression and Purification

Recombinant proteins for biochemical assays were produced in *Escherichia coli* BL21 (DE3) cells, and UTY(KDM6C) for crystallography was produced in Sf9 cells. All proteins were purified by nickel affinity chromatography followed by size exclusion chromatography (Superdex 200). KDM6A and KDM6B were purified as described previously (26, 27).

The UTY(KDM6C) plasmid (Pro^818^–Ser^1347^) was transformed into competent BL21 (DE3) *E. coli* cells and expressed as an N-terminal His_6_-thioredoxin-tagged protein in Terrific Broth medium. When the *A*_600_ reached 0.6, the temperature was dropped to 18 °C, and the culture was induced with 0.2 mm isopropyl β-d-1-thiogalactopyranoside. After 16 h, the cells were harvested and frozen at −80 °C. The thawed pellet was resuspended in 50 mm HEPES, pH 7.5, 500 mm NaCl, 20 mm imidazole, 1 mm DTT, 5% glycerol with 10 μg/ml DNase I with an EDTA-free protease inhibitor tablet (Roche Applied Science). Cells were lysed by sonication, and the lysate was clarified by high speed centrifugation. The lysates were purified using a HisTrap™ HP 5-ml column (GE Healthcare). After loading the lysate, the column was washed with 20 column volumes of 50 mm HEPES, pH 7.5, 500 mm NaCl, 20 mm imidazole, 1 mm DTT, 5% glycerol; protein was then eluted with an imidazole gradient up to 250 mm imidazole over 20 column volumes. The concentrated fractions of protein were further purified using a 300-ml Superdex 200 preparation grade column pre-equilibrated in 50 mm HEPES, pH 7.5, 200 mm NaCl, and 5% glycerol. The purified protein was concentrated, flash-frozen in liquid nitrogen, and stored at −80 °C.

For Sf9 cell expression of UTY(KDM6C) (Leu^878^–Ser^1347^) and UTY(KDM6C) (Ser^840^–Ser^1347^), generation of recombinant baculoviruses, insect cell culture, and infections were performed according to the manufacturer's instructions (Invitrogen). The recombinant proteins were expressed in Sf9 cells, and the cells were collected 72 h postinfection. The cells were resuspended in a buffer containing 50 mm HEPES, pH 7.5, 500 mm NaCl, 10 mm imidazole, 5% glycerol, 0.5 mm tris(2-carboxyethyl)phosphine, 0.4 mm PMSF, 0.64 mm benzamidine, and a proteinase inhibitor mix (Calbiochem), and the protein was purified using nickel affinity chromatography using a stepwise gradient of imidazole. The eluted protein was then incubated with tobacco etch virus protease at 4 °C overnight, followed by size exclusion chromatography (Superdex 200). The tobacco etch virus protease and uncleaved protein were removed using nickel affinity chromatography, and the mass of the cleaved UTY(KDM6C) was verified by electrospray mass ionization time-of-flight mass spectrometry (Agilent LC/MSD). In separate experiments, partially phosphorylated UTY(KDM6C) was treated with λ-phosphatase at a molar ratio of 1:40 simultaneously with the tobacco etch virus protease, and the mass of the dephosphorylated UTY(KDM6C) was verified by electrospray mass ionization time-of-flight mass spectrometry (Agilent LC/MSD). The phosphorylation site was identified by trypsin digestion followed by MS/MS.

##### Crystallization and Data Collection

Crystals of UTY(KDM6C) were obtained using protein expressed and purified from Sf9 cells (Leu^878^–Ser^1347^, isoform 3) using the sitting drop vapor diffusion method at 4 °C by mixing protein and crystallization buffer in a 1:1 ratio in a final volume of 150 nl. Crystals of the UTY(KDM6C)-2OG complex were grown in a drop consisting of 75 nl of protein (9 mg/ml) and 75 nl of a precipitant solution containing 0.1 m HEPES, pH 7.5, 10% (w/v) PEG 3350, and 0.2 m trimethylamine *N*-oxide. To crystallize UTY(KDM6C) with GSK-J1, the protein was preincubated with a 1 mm concentration of the inhibitor. The protein-compound mixture was then transferred to crystallization plates, and crystals were obtained in a drop consisting of 75 nl of protein-compound mix (6.4 mg/ml) and 75 nl of a precipitant consisting of 15% (w/v) PEG 3350, 0.1 m magnesium formate. Crystals from both experiments were cryoprotected with mother liquor supplemented with 25% ethylene glycol before they were flash-frozen in liquid nitrogen. Data sets were collected on beamline I03 and I04-1, respectively, at the Diamond Light source.

A UTY crystal was analyzed using x-ray fluorescence scanning on beamline I02 at Diamond Light Source on a Vortex-EX fluorescence detector (Hitachi High-Technologies Science America Inc.). A peak for zinc (observed peak at 8641.47 eV, expected peak at 8638.9 eV) and a peak for iron (observed peak at 6532.67 eV, expected peak at 6403.8 eV) were observed as expected based on the structural work (see below). No peak for nickel was detected.

##### Structure Determination

The first data set collected for UTY(KDM6C) showed a maximum resolution of 1.80 Å. The data were processed with XDS (28) and were scaled and merged with Aimless (29). Phases were calculated with Phaser within the CCP4 Suite by molecular replacement using KDM6A (PDB code 3AVS) as a search model (30, 31). Solvent flattening was carried out with Parrot to improve density (32), and the initial model was used in Buccaneer to be completed in automated model building (33). The model was further improved by subsequent cycles of model building in Coot (34) and refinement in Refmac5 (35). Although no 2OG had been added during crystallization, density in the active site could be assigned to 2OG during model building. The quality of the structure was assessed with the MolProbity server (36) and deposited in the Protein Data Bank with the PDB code 3ZLI.

The data for the co-crystal structure of UTY(KDM6C) and GSK-J1 showed maximum resolution to 2.00 Å and were processed using XDS (28). Further scaling and merging was done with Aimless (29). Because both crystal forms appeared in identical P 21 21 21 symmetry and with identical cell parameters, the *R*_free_ flag was copied across from the UTY(KDM6C)·2OG data set. The presence of identical cell parameters allowed the calculation of the phases by rigid body refinement in Refmac5 (35), with final *R* of 29.8% and *R*_free_ of 31.2%. The model was then further improved by iterative cycles of building in Coot and refinement with Refmac5 (34, 35). The quality of the structure was assessed with the MolProbity server (36) and deposited in the Protein Data Bank with the PDB code 3ZPO. Data collection and refinement statistics for both structures are given in Table 1.

**TABLE 1:** **Summary of diffraction and refinement statistics**

	UTY(KDM6C) with 2OG (PDB code 3ZLI)	UTY(KDM6C) with GSK-J1 (PDB code 3ZPO)
**Data collection**		
Space group	P 2_1_ 2_1_ 2_1_	P 2_1_ 2_1_ 2_1_
*a*, *b*, *c* (Å); α, β, γ (degrees)	90.58, 110.2, 118.2; 90, 90, 90	91.16, 110.75, 119.49; 90, 90, 90
Wavelength (Å)	0.9763	0.9200
Resolution (Å)*^a^*	49.96–1.80 (1.83–1.80)	39.83–2.00 (2.04–2.00)
*R*_merge_ (%)*^a^*	7.2 (89.6)	11.4 (77.0)
*I*/σ*I**^a^*	13.4 (2.0)	10.4 (2.0)
Completeness (%)*^a^*	100.0 (100.0)	99.1 (97.1)
Redundancy*^a^*	6.7 (6.9)	6.9 (6.1)
**Refinement**		
Resolution (Å)	50.01–1.80	37.50–2.00
No. of reflections	104567	77371
*R*_work_*/R*_free_ (%)	17.60/20.43	17.75/20.98
No. of atoms		
Protein	7118	7134
Ligand/Ion	80	130
Water	549	541
*B*-Factors (Å^2^)		
Protein	A, 31.71; B, 34.20	A, 30.38; B, 31.56
Ligand/Ion	38.55/Zn, 28.26; Fe, 25.00	34.45/Zn, 27.83; Fe, 30.56
Water	38.83	35.75
Root mean square deviations		
Bond lengths (Å)	0.013	0.011
Bond angles (degrees)	1.533	1.39
Ramachandran plot		
Most favored (%)	98.05	97.94
Allowed (%)	1.95	2.06
Disallowed (%)	0.0	0.00

*^a^* Values in parentheses are for the highest resolution shell.

##### Activity Assays

NMR spectroscopy was carried out as described previously (37). NMR spectra were recorded using a Bruker Avance AVIII 700 MHz spectrometer equipped with an inverse TCI cryoprobe, optimized for ^1^H observation, and installed with Topspin 2 software. All samples were prepared in Eppendorf tubes (75-μl volume) before transfer to 2-mm MATCH NMR tubes (Wilgenberg), and time course data were then collected over a period of 50 min at 168-s intervals using an automated routine. The solvent deuterium signal was used as an internal lock signal, and the solvent signal was reduced by presaturation during a 2-s recovery delay. Experiments with histone peptide were monitored using a PROJECT-CPMG (Carr-Purcell Meibbom-Gill) sequence (38), composed of six cycles with τ = 4 ms. The total echo time was 48 ms. Samples were prepared in ammonium formate buffer (dAFN), as described previously (37). Enzyme stocks were in protiated 10 mm HEPES, 200 mm NaCl buffer, pH 7.5, which was diluted with dAFN when added to the samples (Table 2). Chemical shifts are reported relative to tetradeuterotrimethylsilyl propanoic acid (δ_H_ 0.0 ppm), which was either added to the reaction mixture prior to incubation or was used as an external reference.

**TABLE 2:** **Reaction components for NMR analyses** Experiments with UTY variants did not include TSP.

	Stock concentration	Volume	Final concentration
	*mm*	μ*l*	*mm*
Enzyme	0.1	7.5	0.01
2OG	10	3.75	0.5
Ascorbate	10	7.5	1
Fe(II)	1	7.5	0.1
TSP	5.8	1	0.08
H3K27Me_3_ peptide	10	0 or 7.5	0 or 1
dAFN buffer		47.75 or 40.25	

Apparent *K_D_* values were determined by AlphaScreen® assays as described previously (39). Binding assays were carried out as 20-μl reactions in 384-well white ProxiPlates (PerkinElmer Life Sciences) as described (7). His-tagged enzyme (500 nm) was incubated with biotinylated H3K27Me_3_ peptide (Biotin-KAPRKQLATKAARKMe_3_SAPATGG, variable concentration) for 15 min at room temperature in buffer containing 50 mm HEPES (pH 7.5), 0.01% Tween 20, 0.1% BSA. AlphaScreen streptavidin-conjugated donor and nickel chelate-conjugated acceptor beads were added to the wells at a final concentration of 10 μg/ml and incubated for a further 1 h in the dark at 22 °C. The plates were analyzed using an Envision (PerkinElmer Life Sciences) plate reader.

Reactions for MALDI-TOF MS analysis consisted of enzyme (1–10 μm), (NH_4_)_2_Fe(SO_4_)_2_ (10 μm), 2OG (20–500 μm), ascorbate (100 μm), and histone peptide (10–50 μm) in buffer containing 50 mm HEPES, pH 7.5, 50 mm NaCl, 5% glycerol. Reaction components were premixed in two batches ((i) enzyme, Fe(II), and ascorbate and (ii) 2OG and peptide), and reactions were initiated by mixing the enzyme and substrate mix. Reactions were incubated at 37 °C for the indicated times before being quenched with methanol in a 1:1 ratio. Peptide sequences used for screening are given in Table 3.

**TABLE 3:** **Sequences of peptides used for activity screening of UTY(KDM6C)**

Peptide*^a^*	Sequence
H3 K4Me_1_	ARTKMe_1_QTARKSTGGKAPRKQLATKA
H3 K4Me_2_	ARTKMe_2_QTARKSTGGKAPRKQLATKA
H3 K4Me_3_	ARTKMe_3_QTARKSTGGKAPRKQLATKA
H3 K9Me_1_	ARTKQTARKMe_1_STGGKAPRKQLATKA
H3 K9Me_2_	ARTKQTARKMe_2_STGGKAPRKQLATKA
H3 K9Me_3_	ARTKQTARKMe_3_STGGKAPRKQLATKA
H3 K27Me_1_	LATKAARKMe_1_SAPSTGGVKK
H3 K27Me_2_	LATKAARKMe_2_SAPSTGGVKK
H3 K27Me_3_	KAPRKQLATKAARKMe_3_SAPATGG
H3 K36Me_1_	KSAPSTGGVKMe_1_KPHRYRPGTVALRE
H3 K36Me_2_	KSAPSTGGVKMe_2_KPHRYRPGTVALRE
H3 K36Me_3_	KSAPSTGGVKMe_3_KPHRYRPGTVALRE
H4 K20Me_1_	LGKGGAKRHRKMe_1_VLRDNIQGITKPA
H4 K20Me_2_	LGKGGAKRHRKMe_2_VLRDNIQGITKPA
H4 K20Me_3_	LGKGGAKRHRKMe_3_VLRDNIQGITKPA

*^a^* H3, histone 3; H4, histone 4; K, lysine.

For inhibition assays, the same enzyme/Fe(II)/ascorbate mixture was preincubated with 10 μm inhibitor for 15 min before the reaction was initiated by the addition of the peptide substrate solution. Reactions were quenched with 1:1 methanol after 20 min at 37 °C.

Product formation was assessed by MALDI-TOF MS. One microliter of quenched reaction was mixed with 1 μl of α-cyano-4-hydroxycinnamic acid and spotted onto a MALDI-TOF plate for analysis using a MALDI micro-MX mass spectrometer (Waters) in reflectron positive ion mode with pulse voltage 1250 V, detector voltage 2750 V, and mass suppression 1000 Da. Data were analyzed using MassLynx version 4.0. For inhibitor assays, inhibition levels were measured relative to an inhibitor-free reaction.

##### Cell Culture, Immunofluorescence, and Dual Luciferase Assays

For cell-based experiments, HEK 293T and HeLa cells were cultured at 37 °C and 5% CO_2_ in DMEM (Lonza) supplemented with 10% fetal bovine serum (FBS) (Invitrogen) and 1% Glutamax (Invitrogen). For microscopy, HeLa cells were seeded into 96-well clear bottom optical grade plates (BD Biosciences) at 8000 cells/well and allowed to settle overnight. Transfections were carried out the next day. For each well, 0.1 μg of plasmid and 0.25 μl of Lipofectamine 2000 (Invitrogen) were each incubated in 25 μl of Opti-MEM (Invitrogen) for 5 min at room temperature. The two solutions were then mixed gently but thoroughly and incubated for 20 min at room temperature. The mixture was added to the cells in 50 μl of fresh medium. Four hours later, the medium was changed, and the cells were incubated for a further 24 h before fixation. Cells were fixed in 4% (v/v) aqueous formaldehyde for 20 min, permeabilized in 0.5% Triton X-100 in phosphate-buffered saline (PBS) for 8 min, and blocked in 3% (w/v) FBS in PBS for 1 h with a PBS wash between each step. The primary antibodies were then added to the cells in blocking solution. After 1 h at room temperature, cells were washed three times in PBS, incubated with secondary antibodies for 1 h, rinsed three times in PBS again, and counterstained with DAPI before imaging. Luciferase activity assays were carried out as described previously (13, 22, 40). The atrial natriuretic factor (ANF) promoter-luciferase reporter was a kind gift from Benoit Bruneau (41). As for immunofluorescence assays, this construct was co-transfected into HEK 293T cells in the presence of full-length (FL) WT UTY(KDM6C) or H1095 UTY(KDM6C). Luciferase activity was measured using the Promega Dual-Luciferase Reporter Assay System on a Promega GloMax 20/20 luminometer. Readings were normalized to a *Renilla* luciferase control that was co-transfected with all samples.

## RESULTS

**FIGURE 2.**
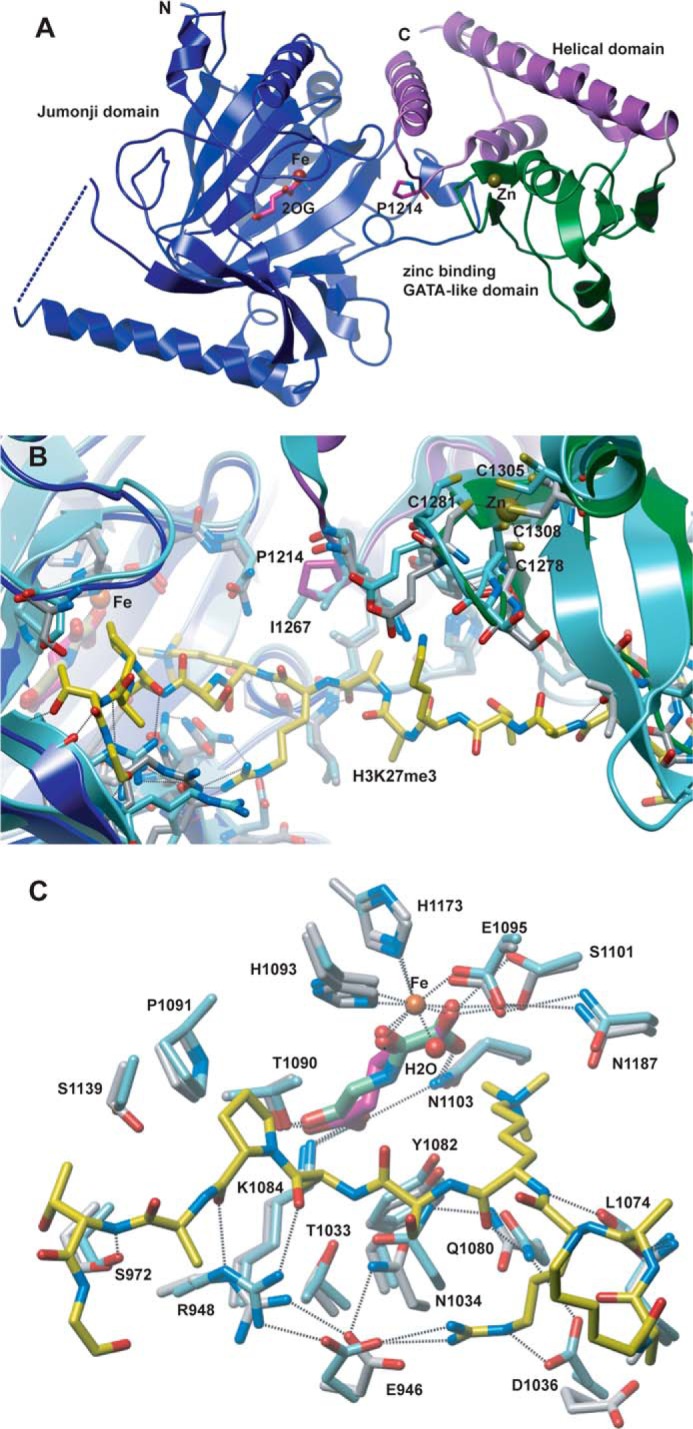
**Views from a crystal structure of UTY(KDM6C).** *A*, overall structure of UTY(KDM6C)(882–1344) (PDB code 3ZLI) in complex with 2OG (*pink*), Fe(II) (*orange*), and Zn(II) (*yellow*) showing the jumonji (*blue*), helical (*purple*), and zinc binding (*green*) domains. The mutated residue Pro^1214^ that renders UTY(KDM6C) more active as an H3K27Me_3_ demethylase is shown as *sticks* in *purple. B*, UTY(KDM6C) *colored* as in *A*, superimposed with KDM6A (*cyan*, PDB code 3AVR) in complex with a H3K27Me_3_ peptide (*yellow*). The residues in KDM6A that interacts with the histone peptide are conserved within UTY(KDM6C), with the exception of Ile^1267^ (*cyan*) that in UTY(KDM6C) is replaced by Pro^1214^ (*purple*). *C*, comparison of 2OG (*pink*)/NOG (*green*) coordination in the UTY(KDM6C) (*silver*) and KDM6A (*cyan*, PDB code 3AVR) structures. The coordinating residues in UTY(KDM6C) are *numbered*. Fe(II) (or surrogate metal; *orange*) and the corresponding coordinated water molecule (*red*) are *spheres*. The H3K27Me_3_ peptide in the KDM6A structure (PDB code 3AVR) is shown as *yellow sticks*.

Studies on the KDM4/JMJD2 KDMs reveal that substitutions in the immediate vicinity of the iron-binding active site can substantially alter substrate selectivity and KDM activity (42). Thus, we initially carried out crystallographic studies on the catalytic domain of UTY(KDM6C) to investigate whether UTY(KDM6C) has a three-dimensional structure similar to that of KDM6A/B. A crystal structure of the C-terminal JmjC and zinc-binding domains of UTY(KDM6C) (Leu^878^–Ser^1347^, isoform 3) was solved to 1.8 Å resolution in complex with its co-substrate 2OG (PDB code 3ZLI). As observed for KDM6B and KDM6A, the UTY(KDM6C) structure reveals three conserved domains: the JmjC domain (residues 881–1188, *blue* in Fig. 2), a linker helical domain (residues 1193–1258 and 1327–1344, *purple* in Fig. 2) and a zinc-binding domain (residues 1263–1326, *green* in Fig. 2). The first helix in KDM6A and the linker region (residues 886–902 in KDM6A) that connects the helical and JmjC domains are not observed in our current crystal structure of UTY(KDM6C). Superimposition of the UTY(KDM6C) structure with its X-linked paralog KDM6A (PDB code 3AVS) shows that the secondary and tertiary folds are almost identical (backbone root mean square deviation value at C_α_ = 0.93), with only minor differences being observed in the loops in the zinc-binding domain.

The UTY(KDM6C) JmjC domain contains 13 β-strands and 10 helices, including the extended and distorted double-stranded β-helix that is characteristic of the 2OG oxygenase superfamily (3, 43). Although there are minor differences in the active site region, overall the KDM6B, KDM6A, and UTY(KDM6C) JmjC folds are very similar. At the active site, the nature of coordination by the metal binding residues is also well conserved, with metal binding by the *N*^ϵ^-imidazole nitrogens of His^1093^ and His^1173^ and by the carboxylate of Glu^1095^ (Fig. 2*C*). Analysis of the electron density at the active site supports presence of 2OG in the cofactor binding pocket. 2OG coordinates the active site metal in a bidentate manner via its oxalyl group, with a water-ligated coordination site adjacent to the predicted location of the substrate *N*^ϵ^-methylated lysine group (as observed in KDM6B and KDM6A enzyme-substrate complexes). Notably, the unusual (within structurally characterized 2OG oxygenases) presence of two cysteinyl residues (Cys^1111^ and Cys^1181^) in the 2OG binding pocket is observed in UTY(KDM6C) as in other KDM6 subfamily members (25, 30).

The UTY(KDM6C) zinc-binding domain is also similar to those in KDM6A/B, with minor differences being observed in loops linking the zinc-binding residues. This domain has been shown to be important for substrate binding in KDM6A (30). In each of the KDM6 subfamily members' zinc-binding domains, the Zn(II) is coordinated by four cysteines, Cys^1278^, Cys^1281^, Cys^1305^, and Cys^1308^, in UTY(KDM6C) (Fig. 2*B*).

Comparison of the structures reveals that the Fe(II)/2OG binding sites are highly conserved in all human KDM6 members. We therefore analyzed the crystallographically observed modes of substrate binding in KDM6A/B to investigate the reported lack of activity with UTY(KDM6C), using the KDM6B (PDB code 4EZH) (25) and KDM6A (PDB code 3AVR) (30) substrate complexes. Superposition of the three structures implies that most of the hydrogen-bonding interactions in KDM6A/B substrate binding probably occur in UTY(KDM6C). Notably, however, one residue, located in the loop between the JmjC and helical domains and which in KDM6A (Ile^1267^) and KDM6B (Ile^1511^) appears to form hydrophobic interactions with Ala^25^ of the H3K27Me_3_ substrate, is substituted for a prolyl residue (Pro^1214^) in UTY(KDM6C) (see below).

**FIGURE 3.**
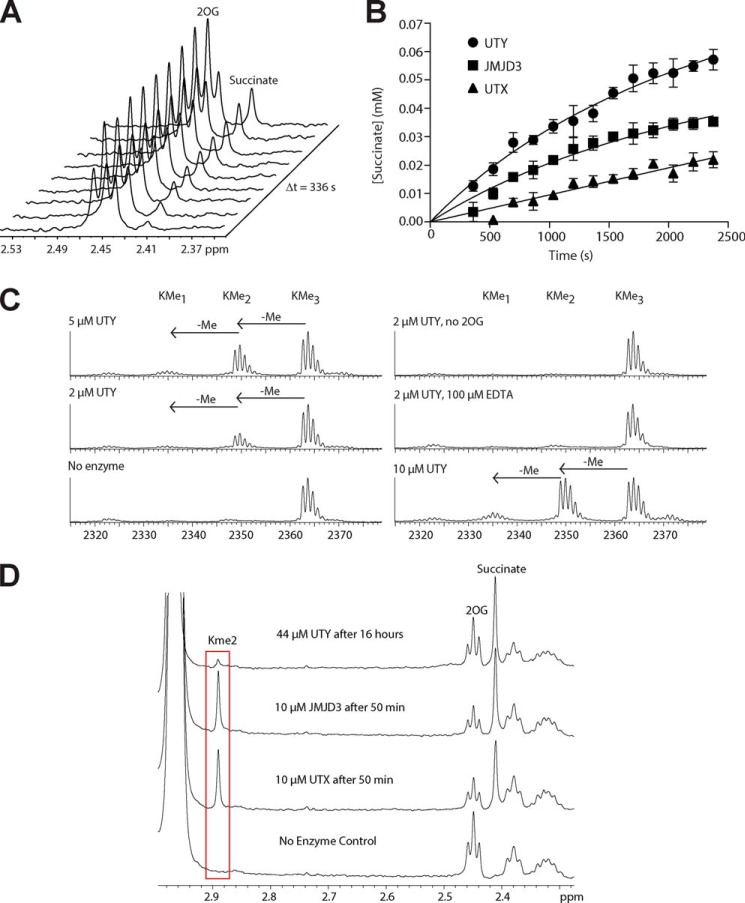
**Purified UTY(KDM6C) is an active *N*^ϵ^-methylated histone 3 lysine 27 demethylase.** *A*, UTY(KDM6C)(818–1347) catalyzes turnover of 2OG to succinate in the absence of prime substrate as measured by ^1^H NMR spectroscopy. *B*, rate of succinate production from prime substrate uncoupled 2OG turnover by UTY, JMJD3, and UTX, as measured by ^1^H NMR spectroscopy. *C*, UTY(KDM6C) catalyzes the demethylation of a 23-residue histone peptide containing H3K27Me_3_ (GGKAPRKQLATKAARKMe_3_SAPATGG), as measured by MALDI mass spectrometry. *D*, KDM6 catalyzed demethylation of a 24-residue histone peptide containing H3K27Me_3_ (GGKAPRKQLATKAARKMe_3_SAPATGGV) measured by ^1^H NMR spectroscopy. *Error bars*, S.D.

Given the observed structural similarity between the UTY(KDM6C) structure and the previously solved KDM6A structures (PDB entries 3AVR and 3AVS) (30), we were interested in revisiting studies on UTY(KDM6C) biochemical activity (7, 9). 2OG oxygenases couple the oxidative decarboxylation of 2OG to oxidation of their prime substrate (Fig. 1*C*). In most cases, 2OG decarboxylation can also occur in the absence of the “prime” substrate (termed “uncoupled turnover”), albeit at reduced levels (5). We produced recombinant UTY(KDM6C) in both *E. coli* (residues 818–1347, corresponding to isoform 3) and Sf9 cells (residues 887–1347 and 840–1347, isoform 3) and looked for evidence of uncoupled activity that would be indicative of the enzyme being correctly folded and catalytically active, using NMR spectroscopy to observe conversion of 2OG to succinate. The ^1^H NMR analyses indicated consumption of 2OG (decrease in the triplet resonance at δ_H_ = 2.45 ppm) and production of succinate (formation of a singlet resonance at δ_H_ = 2.41 ppm), indicating that all of our variants of recombinant UTY(KDM6C) were active (Fig. 3*A*). The rate of uncoupled 2OG turnover of UTY(KDM6C) was found to be comparable with that observed for both recombinant KDM6A and KDM6B (Fig. 3*B*).

With catalytically active UTY(KDM6C) in hand, we then investigated whether a longer UTY(KDM6C) construct (residues 818–1347, herein UTY(KDM6C)) can act as a KDM. UTY(KDM6C) was incubated for 1 h at 37 °C with histone peptide fragments corresponding to known mono-, di-, and tri- lysine methylation sites of histone 3 (at Lys^4^, Lys^9^, Lys^27^, and Lys^36^) and histone 4 (at Lys^20^) (Table 3). Demethylation was assessed by a mass spectrometric (MS) assay with mass shifts of −14 and −28 Da, corresponding to mono- and didemethylation, respectively. No demethylation was observed for histone 3 Lys^4^, Lys^9^, and Lys^36^ or histone 4 Lys^20^ *N*^ϵ^-methylated peptides (as for KDM6B and KDM6A) (6, 9, 10). However, as for the positive controls using the other subfamily members, KDM6A/B, demethylation was observed for the H3K27Me_3_ peptide (histone 3, 12–34) (Fig. 3*B*). The predominant product was H3K27Me_2_, resulting from a single demethylation; a smaller amount of H3K27Me_1_ was observed as a result of didemethylation (Fig. 3*C*). Demethylation was dependent on the presence of Fe(II)/2OG. UTY(KDM6C)-catalyzed demethylation was confirmed by NMR spectroscopy, where formation of a dimethyllysine peak in the ^1^H NMR spectrum was observed at δ_H_ = 2.89 ppm (Fig. 3*D*).

Phosphorylation sites are reported on many JmjC KDMs (44, 45). During characterization of UTY(KDM6C) produced in insect cells by MS analysis, phosphorylation of UTY(KDM6C) Thr^887^ was identified. Thr^887^ is conserved in the KDM6 subfamily and located in the N-terminal part of the JmjC domain, away from cofactor/predicted peptide binding sites. Because the JmjC histone KDM PHF2 is reported only to be active on phosphorylation of Ser^1056^ (45), we tested if a lack of phosphorylation could explain the low KDM activity of UTY(KDM6C) from *E. coli*. However, no significant difference in 2OG turnover, as judged by ^1^H NMR spectroscopy, was detected between partially phosphorylated UTY(KDM6C) and protein dephosphorylated *in vitro* using λ-phosphatase (both enzymes (10 μm) produced 7 ± 0.5 nmol of succinate in 28 min).

**FIGURE 4.**
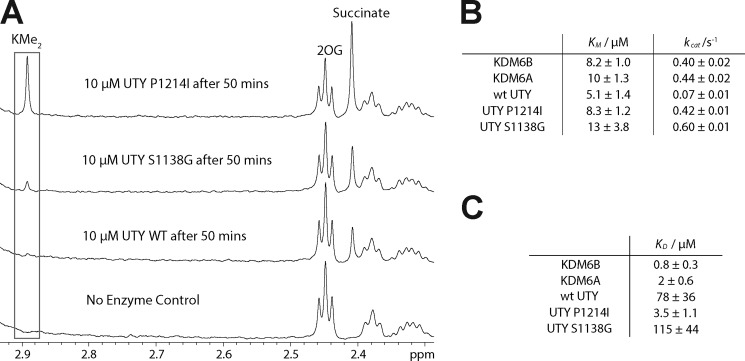
**A point variant of UTY(KDM6C) is more active than WT UTY(KDM6C).** *A*, UTY(KDM6C) P1214I is a more active KDM than WT UTY(KDM6C) and UTY(KDM6C) S1138G. Demethylation of UTY(KDM6C) and UTY(KDM6C) variants was measured by NMR spectroscopy. *B*, apparent 2OG *K_m_* and *k*_cat_ values for all members of the KDM6 subfamily and UTY(KDM6C) variants are similar. 2OG *K_m_* and *k*_cat_ values were measured by a MALDI mass spectrometry coupled assay. *C*, apparent H3K27Me_3_ peptide *K_D_* values for all members of the KDM6 subfamily and UTY(KDM6C) variants. UTY(KDM6C) binds peptide more weakly than KDM6B and KDM6A. UTY(KDM6C) P1214I shows a tighter binding, similar to KDM6B and KDM6A rather than WT UTY(KDM6C). *K_D_* values were measured by an ALPHA screen-based assay.

The catalytic domains of UTY(KDM6C) and KDM6A share 88% sequence identity (Fig. 1*A*). Despite this, the demethylation activity of UTY(KDM6C) appears to be much reduced relative to KDM6A, as measured by NMR (Fig. 3*D*). Using sequence alignments, we identified two residues, Ser^1138^ and Pro^1214^, that differ significantly from those present in KDM6A/B (where the equivalent residues are glycine and isoleucine, respectively). We proposed that these residues could be important in peptide binding on the basis of comparison of the UTY(KDM6C) structure with that of a recently reported co-crystal structure of KDM6A with an H3K27Me_3_ peptide (PDB code 3AVR) (30). We therefore converted the Ser^1138^ and Pro^1214^ residues of UTY(KDM6C) individually to the residues present within KDM6A/B to give two variants, UTY(KDM6C) S1138G and UTY(KDM6C) P1214I, which were produced in recombinant form in *E. coli*. Although UTY(KDM6C) S1138G showed activity levels similar to those of WT UTY(KDM6C), UTY(KDM6C) P1214I was considerably more active by NMR (Fig. 4*A*). 2OG *K_m_* values for all of the KDM6 family and the UTY(KDM6C) variants were determined using an MS assay and a 23-residue H3K27Me_3_ peptide substrate. 2OG *K_m_* values were similar for all five enzymes/variants (Fig. 4*B*). *k*_cat_ values, however, indicated that WT UTY(KDM6C) and UTY(KDM6C) S1138G were considerably less active than UTY(KDM6C) P1214I, which showed turnover rates similar to that of KDM6A/B. The UTY(KDM6C) activity was too low to measure accurate peptide substrate *K_m_* values. Thus, an AlphaScreen® assay was used to measure apparent binding constants (*K_D_*) with an H3K27Me_3_ peptide (39). The results showed UTY(KDM6C) to have a higher substrate *K_D_* value than KDM6A/B (78 μm *versus* 0.8/2 μm for KDM6B/A), with UTY(KDM6C) P1214I showing a *K_D_* value similar to KDM6B/A (3.5 μm), consistent with this variant's higher enzymatic activity (Fig. 4*C*). This suggests that the isoleucyl residue present in KDM6A and KDM6B, but not UTY(KDM6C), provides important interactions for peptide binding.

**FIGURE 5.**
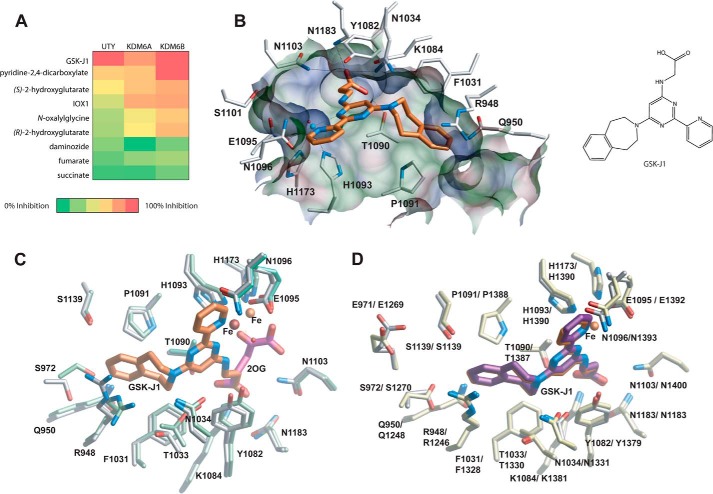
**UTY(KDM6C) shows a similar inhibitor profile to KDM6A and KDM6B.** *A*, KDM6B, KDM6A, and UTY(KDM6C) show similar responses to a panel of 2OG oxygenase inhibitors. A panel of nine inhibitors were ranked by their potency at 10 μm. IOX1 is 5-carboxy-8-hydroxyquinoline. *B*, *left*, molecular interactions between a KDM6 subfamily inhibitor GSK-J1 (*orange*) and residues within the catalytic pocket of UTY(KDM6C) (*silver*) (25). Hydrogen bonds are shown as *dashed lines. Right*, structure of GSK-J1. *C*, overlay of the structures of UTY(KDM6C)(882–1344) in complex with 2OG (*pink*) or GSK-J1 (*orange*). The residues within the catalytic pocket of the UTY(KDM6C)-2OG complex (PDB code 3ZLI) are *green*, and iron is shown as an *orange sphere*. In the UTY(KDM6C)·GSK-J1 complex (PDB code 3ZPO), residues within the catalytic pocket are *silver*, and the iron is shown as a *brown sphere. D*, comparison of GSK-J1 binding in UTY(KDM6C) and KDM6B. In the UTY(KDM6C)·GSK-J1 complex (PDB code 3ZPO), residues within the catalytic pocket are *silver*, and GSK-J1 is shown in *orange*. In the KDM6B·GSK-J1 complex (PDB code 4ASK) (1), residues within the catalytic pocket are *yellow*, and GSK-J1 is shown in *purple*. The iron is shown as an *orange sphere*. Note the identical binding modes of GSK-J1 in the two structures (within error).

The JmjC KDMs share a common distorted jelly-roll (or double-stranded β-helix) fold with all other 2OG oxygenases, and all use 2OG as a co-substrate (3, 43). Most known KDM inhibitors are competitive with respect to 2OG, and as such, their inhibitor profiles can be similar; however, it is becoming increasingly evident that it is possible to identify inhibitors that discriminate between different subfamilies (26). Recently a small molecule inhibitor of KDM6A/B, GSK-J1, has been described (25). To ascertain whether UTY(KDM6C) showed an inhibitor profile similar to those of the other KDM6 subfamily members, a set of nine known KDM inhibitors were selected, and the response of KDM6A, UTY(KDM6C), and KDM6B to them (at 10 μm) was assessed, by measuring inhibition of demethylation of an H3K27Me_3_ peptide (25, 26, 40, 46). All three KDMs displayed similar inhibition profiles, and, as anticipated, given its similarity to KDM6A, inhibition of UTY(KDM6C) activity by GSK-J1 was observed (Fig. 5*A*). GSK-J1 was the most potent inhibitor of KDM6B, KDM6A, and UTY(KDM6C) of the nine inhibitors tested. We were therefore interested to see whether GSK-J1 bound in a similar mode in UTY(KDM6C) as it does in KDM6A and KDM6B and attempted co-crystallization of UTY(KDM6C) with GSK-J1; co-crystals were grown, and the UTY(KDM6C)·GSK-J1 complex structure was solved to 2.0 Å resolution (PDB code 3ZPO).

A structure of UTY(KDM6C) with GSK-J1 reveals that GSK-J1 is bound to the 2OG binding site identically within error to that observed with KDM6B·GSK-J1 complex (Fig. 5, *B–D*) (25); the propanoic acid of GSK-J1 is positioned to hydrogen-bond with Asn^1103^, Lys^1084^, and Thr^1090^, and the phenyl ring of the terahydrobenzazepine, as in the KDM6B·GSK-J1 complex, is sandwiched in a hydrophobic cleft between Arg^948^ and Pro^1091^. The pyridyl-pyrimidine biaryl heterocyclic GSK-J1 ring system is positioned to make bidentate interactions with the iron, and similarly to KDM6B, it translocates the iron ∼2.4 Å away from the H*X*E … H triad. Such metal movement has been observed with other 2OG oxygenases with certain iron-chelating inhibitors and may, in some circumstances, reflect potent inhibition (25, 40).

**FIGURE 6.**
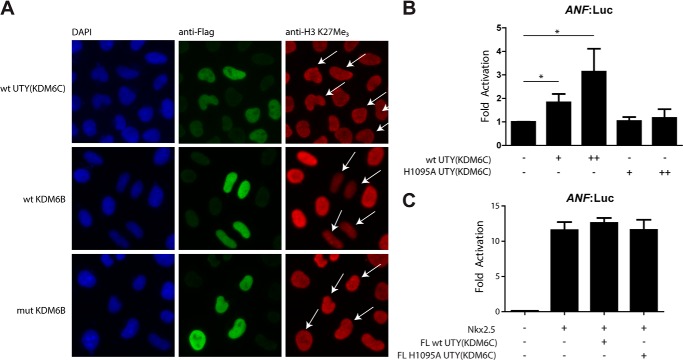
**Activity of UTY(KDM6C) in cells.** *A*, immunofluorescence studies indicate no change in H3K27Me_3_ levels upon overexpression of WT UTY(KDM6C). FL WT UTY(KDM6C), FL WT KDM6B, and a FL catalytic mutant of KDM6B were exogenously expressed in HeLa cells. Nuclei were stained with DAPI (*blue*), cells containing overexpressed enzyme were stained with an anti-FLAG antibody (*green*), and the level of H3K27Me_3_ was quantified with an antibody to this mark (*red*). Cells overexpressing enzyme in the anti-H3K27Me_3_ staining are identified by *white arrows. B*, dual luciferase assay measuring the expression of luciferase from the *ANF* promoter in the presence of FL WT and H1095A UTY(KDM6C) in HEK 293T cells shows enhanced expression when wild type UTY(KDM6C) is present. Experiments show the average of three biological replicates of triplicate transfections. *, *p* < 0.05 in an unpaired Student's *t* test. *C*, dual luciferase assay measuring the expression of luciferase from the *ANF* promoter in the presence of Nkx2.5 in HEK 293T cells shows enhanced expression from the ANF promoter. The addition of either FL WT or H1095A UTY(KDM6C) does not further enhance expression from the *ANF* promoter. *Error bars*, S.D.

Having shown that UTY(KDM6C) is active *in vitro*, we were interested in investigating its activity in cells. An immunofluorescence-based assay was used to investigate global changes in H3K27Me_3_ levels in HeLa cells on overexpression of FL UTY(KDM6C) or the likely catalytically inactive FL H1095A mutant UTY(KDM6C) (which is missing one Fe(II)-binding residue). As observed previously, exogenous expression of FL WT UTY(KDM6C) caused no detectable decrease in the global levels of H3K27Me_3_ (Fig. 6*A*), although a decrease was observed in the positive control with FL WT KDM6B, but not with the catalytically inactive KDM6B variant (7, 9). These results suggest that the demethylase activity of UTY(KDM6C) may be limited to specific targets (possibly including non-histone substrates)/contexts or that observation of its activity is obscured by the greater activity of KDM6A (which is also present endogenously in HeLa cells), such that UTY(KDM6C) KDM activity is not observed as a global change (see “Discussion”).

Recently, KDM6A has been shown to be involved in control of cardiac gene expression and to be crucial in heart development (13, 22). Using a dual luciferase assay measuring expression of luciferase driven by the atrial natriuretic factor *(ANF)* promoter in HEK 293T cells in the presence of exogenously overexpressed KDM6A and the transcription factor Nkx2.5, enhanced *ANF* expression is also observed (22). Shpargel *et al.* (13) report that increased *ANF* expression is also observed with overexpression of UTY(KDM6C) but to a lesser effect, which was proposed to be due to a non-catalytic/demethylation mechanism. We overexpressed FL WT UTY(KDM6C) and FL catalytically inactive H1095A UTY(KDM6C) in addition to a luciferase-producing plasmid in HEK 293T cells. Overexpression of full-length WT UTY(KDM6C) caused a 5-fold increase in *ANF* promoter-coupled luciferase expression, relative to overexpression of an empty vector control, which is not observed with exogenous expression of the full-length catalytically inactive H1095A UTY(KDM6C) mutant (Fig. 6*B*). These results suggest that UTY(KDM6C) could have a catalytic function in cells, although they do not directly link it to histone demethylase activity. In contrast to previous studies, we did not observe a further enhancement in *ANF* expression when UTY(KDM6C) was expressed together with the transcription factor Nkx2.5 (Fig. 6*C*).

## DISCUSSION

Non-catalytically active homologues or isoforms of enzymes that catalyze covalent reactions have attracted recent attention (47). From a functional perspective, it is clear that many of these “inactive” enzymes have non-catalytic functions within the cell; they can bind to the same substrates as their active homologues (hence inhibiting binding of “active” enzymes), they can help with targeting of other components, or they can bind to receptors, thereby activating signaling pathways (48, 49). Thus, in one scenario, UTY(KDM6C) is a closely related homologue of KDM6A/B that, at least in principal, can act in a regulatory manner via non-covalent interactions with nucleosomes involving its JmjC catalytic domain. It may exert a regulatory role via mechanisms, including competition with other histone-binding or -modifying proteins, including KDMs, such as KDM6B and KDM6A.

However, our work clearly demonstrates that UTY(KDM6C) is a catalytically active KDM6 family member. Although UTY(KDM6C) has reduced levels of activity with respect to H3K27Me_3_ substrates when compared with KDM6A/B *in vitro*, it has fully competent Fe(II)/2OG binding sites and unequivocally has KDM activity, as shown by NMR and MALDI-TOF MS experiments (Fig. 3). Further, crystal structures of the JmjC and Zn(II) domains of UTY(KDM6C) demonstrate that binding to both 2OG and the inhibitor, GSK-J1, displays identical structure to KDM6A/B (Figs. 2 and 5, *B–D*). Thus, UTY(KDM6C) is a catalytically functional KDM. It is notable that although UTY(KDM6C) has relatively low activity with an H3K27 peptide fragment substrate, its activity can be increased by substitution of a single residue (P1214I), which promotes substrate binding. It is thus quite possible that as yet unidentified factors will enable increased UTY(KDM6C) activity in cells, simply by increasing the strength of substrate binding. *In vivo* it is also possible that UTY(KDM6C) may show enhanced activity with a multiply modified histone tail. Alternatively, it is possible that the apparently inefficient KDM activity of UTY(KDM6C) is related to its, as yet incompletely defined, physiological role. In this regard, it is of interest that the slow reaction of some 2OG oxygenases with respect to oxygen is proposed to help to enable them to act as hypoxia sensors (50, 51).

It is also conceivable that the KDM activity of UTY(KDM6C) (and indeed some other KDMs) is largely unrelated to its main role in transcriptional regulation (see below) (*e.g.* as observed in the interaction of T box transcription factors with KDM6B) (18). However, the observation that the catalytic machinery common to all JmjC enzymes is intact in UTY(KDM6C) and that a single substitution, Pro^1214^, significantly enhances the activity of recombinant human UTY(KDM6C) to levels comparable with KDM6A and KDM6B *in vitro* argues against this proposal. In other cases where catalytically inactive isoforms have apparently evolved from active ones, the substituted residue(s) tend to be crucial for catalysis. For example, in the 10% of kinase homologues predicted not to have a catalytic function, at least one of three residues known to be essential for catalysis is missing (52). If the catalytic activity of UTY(KDM6C) is (largely) independent of its biological role, one might have expected loss of the essential Fe(II) and 2OG binding sites (*e.g.* as is likely to occur for the KDM homologue JARID2, which is missing two of the required Fe(II)-binding residues and is currently assigned to be catalytically inactive (53)). Thus, it seems likely that the KDM activity of UTY(KDM6C) is related to its biological function.

The increased KDM activity of UTY(KDM6C) P1214I relative to WT UTY(KDM6C) (at least with the tested histone fragments) and the tighter peptide binding interaction of this variant suggest that hydrophobic interactions between this isoleucyl residue in KDM6A and KDM6B and Ala^25^ of the histone 3 tail are important for histone binding and enzymatic activity. A recent study demonstrates that the reverse mutation in KDM6A (I1267P)/KDM6B (I1509P) reduces, but does not abolish, KDM activity, as assayed by immunofluorescence in cells, consistent with this proline to isoleucine substitution not being the only difference between UTY(KDM6C) and KDM6A/B responsible for the reduced activity (13). Interestingly, Shpargel *et al.* (13) show that a further substitution, present in mouse, but not human, UTY(KDM6C) abolishes global reduction of H3K27Me_3_ by UTY(KDM6C) using an immunofluorescence-based assay, although they do not investigate activity on peptidic substrates. All three of the KDMs, UTY(KDM6C), KDM6A, and KDM6B, possess additional tetratricopeptide repeat domains (Fig. 1*B*), which probably also contribute indirectly to substrate binding and, in cells, may outweigh the loss in binding interaction from these single amino acid substitutions. Like most, if not all, chromatin-modifying enzymes, both UTY(KDM6C) and KDM6A will also operate in multicomponent complexes, and it is possible that additional co-factors are required for optimal demethylase activity.

It is possible that UTY(KDM6C) acts on substrates other than histones; indeed, there is increasing evidence that lysine methylation/demethylation of non-histone proteins, at present to a lesser extent than for histones, play important roles in multiple regulatory pathways, including with p53 and NF-κB (54, 55). In the case of one 2OG oxygenase involved in transcriptional regulation, there is extensive evidence that it acts on multiple substrates. Factor inhibiting HIF (FIH), which like the 2OG KDMs is a JmjC enzyme, catalyzes asparaginyl hydroxylation in the C-terminal transactivation domain of hypoxia-inducible factor (HIF), a modification directly regulating HIF-mediated transcription by blocking binding to transcriptional coactivators (56, 57). In addition to HIF, FIH has multiple ankyrin repeat protein substrates for which the role of hydroxylation is unknown (58). Thus, it is possible that, as for FIH, the physiological role(s) of UTY(KDM6C) (and other KDMs) involves the interaction of multiple proteins, only some of which are efficient substrates at the active site of the catalytic domains.

In conclusion, we have provided definitive biochemical evidence that purified UTY(KDM6C) can act as a KDM. Further studies are required to elucidate the full role of UTY(KDM6C) catalyzed demethylation in cells. As in previous studies, we were unable to observe a decrease in global levels of H3K27Me_3_ levels upon overexpression of UTY(KDM6C) in HEK 293T cells. However, dual luciferase assays show an enhancement in *ANF* promoter-linked luciferase expression, which is dependent on the catalytic activity of UTY(KDM6C). This suggests that UTY(KDM6C) may activate transcription in a gene-specific manner, which would not be observed in global analyses of histone modifications. KDM6A and KDM6B have also been shown to be involved in *HOX* gene regulation during development. Notably, within KDMs, JMJD1A has recently been shown to alter expression levels of the *SRY* gene, regulating sex determination (59). Given the ability of UTY(KDM6C) to demethylate H3K27Me_3_ and thus activate repressed genes, UTY(KDM6C) may be required in male sex determination during development.

## Footnotes

3 The abbreviations used are:

KDM: lysine demethylase
JmjC: Jumonji C
2OG: 2-oxoglutarate
H3K27: histone 3 lysine 27
H3K27Me: methylated H3K27
PDB: Protein Data Bank
ANF: atrial natriuretic factor
FL: full-length
WT: wild type
HIF: hypoxia-inducible factor
FIH: factor inhibiting HIF.
